# Arabidopsis fibrillin 1-2 subfamily members exert their functions via specific protein–protein interactions

**DOI:** 10.1093/jxb/erab452

**Published:** 2021-10-15

**Authors:** Diego Torres-Romero, Ángeles Gómez-Zambrano, Antonio Jesús Serrato, Mariam Sahrawy, Ángel Mérida

**Affiliations:** 1 Instituto de Bioquímica Vegetal y Fotosíntesis, Consejo Superior de Investigaciones Científicas (CSIC) – Universidad de Sevilla, Avenida Américo Vespucio 49, 41092 Sevilla, Spain; 2 Estación Experimental del Zaidín, CSIC, Calle Profesor Albareda 1, 18008 Granada, Spain; 3 Boyce Thompson Institute. USA

**Keywords:** Abiotic stress, Arabidopsis, fibrillin, jasmonate, photosystem II, plastoglobule

## Abstract

Fibrillins (FBNs) are plastidial proteins found in photosynthetic organisms from cyanobacteria to higher plants. The function of most FBNs remains unknown. Here, we focused on members of the FBN subgroup comprising FBN1a, FBN1b, and FBN2. We show that these three polypeptides interact between each other, potentially forming a network around the plastoglobule surface. Both FBN2 and FBN1s interact with allene oxide synthase, and the elimination of any of these FBNs results in a delay in jasmonate-mediated anthocyanin accumulation in response to a combination of moderate high light and low temperature. Mutations in the genes encoding FBN1s or FBN2 also affect the protection of PSII under the combination of these stresses. Fully developed leaves of these mutants have lower maximum quantum efficiency of PSII (*F*_v_/*F*_m_) and higher oxidative stress than wild-type plants. These effects are additive, and the *fbn1a-1b-2* triple mutant shows a stronger decrease in *F*_v_/*F*_m_ and a greater increase in oxidative stress than *fbn1a-1b* or *fbn2* mutants. Co-immunoprecipitation analysis indicated that FBN2 also interacts with other proteins involved in different metabolic processes. We propose that these fibrillins facilitate accurate positioning of different proteins involved in distinct metabolic processes, and that their elimination leads to dysfunction of those proteins.

## Teaser

Members of the FBN1-2 subfamily in Arabidopsis interact with each other and with other proteins. These interactions may explain the pleiotropic effect of mutations in the genes encoding these proteins.

## Introduction

Plastoglobules (PGs) are lipoprotein bodies present in all types of plant plastids. In chloroplasts, they are attached to thylakoids through a half-lipid bilayer that surrounds the globule contents and is continuous with the stroma-side leaflet of the thylakoid membrane (Austin *et al.*, 2006). The lipids of chloroplast PGs consist mainly of prenylquinones and triacylglycerol, and PGs have been proposed to serve as lipid microcompartments for the synthesis, storage, and redistribution of these subsets of isoprenoids and neutral lipids (van Wijk and Kessler, 2017). However, proteomic analysis has revealed the presence of different proteins in PGs (Vidi *et al.*, 2006; Ytterberg *et al.*, 2006). Lundquist *et al.* (2012) defined a core PG proteome core consisting of 30 proteins, which were classified into four modules using a genome-wide co-expression analysis: module 1 (senescence, chlorophyll degradation, proteolysis), module 2 (plastid carotenoid metabolism, plastid proteolysis), module 3 (redox regulation, photoacclimation), and module 4 (plastid biogenesis and the Calvin–Benson cycle). These studies demonstrated that PGs are not a simple lipid reservoir and that proteins involved in different metabolic processes of the chloroplast are associated with them. The most abundant proteins in PGs include fibrillins (FBNs) and six ABC1 kinases, constituting 53% and 19% of the core PG proteome mass, respectively (Lundquist *et al.*, 2012). FBNs constitute a large protein family present in photosynthetic organisms from cyanobacteria to higher plants (Laizet *et al.*, 2004). They are referred to as fibrillins because they were first identified in fibrils, the thread- or tube-like structures found in bell pepper (*Capsicum annuum*) fruit chromoplasts (Deruere *et al.*, 1994). These fibrils are the main sites for pigment accumulation in chromoplasts (Winkenbach *et al.*, 1976) The FBNs in higher plants and algae can be divided into 12 clades (Singh and McNellis, 2011). Seven of them are located in PGs and form part of its core proteome (FBN1a, FBN1b, FBN2, FBN4, FBN7a, FBN7b, and FBN8), and the rest have a stromal or thylakoid-associated location (Lundquist *et al.*, 2012). FBNs have been proposed to be involved in central processes of plant physiology, particularly those involved in plant responses to stress and plastid architectural development (Singh and McNellis, 2011). However, their mechanisms of action have not yet been elucidated. Recently, the stroma-localized FBN5 was shown to be essential for plastoquinone-9 biosynthesis by binding to solanesyl diphosphate synthases in Arabidopsis (Kim *et al.*, 2015), and the elimination of FBN6 led to perturbation of reactive oxygen species (ROS) homeostasis (Lee *et al.*, 2020). FBN1a and FBN1b arose from a recent duplication event on chromosome 4 and, together with FBN2, they form a subgroup within the FBN family. Most of the FBNs characterized to date in different species belong to this subfamily (Laizet *et al.*, 2004) and, together with FBN4, they constitute the most abundant proteins in PGs (Lundquist *et al.*, 2012). A bell pepper orthologue of Arabidopsis FBN1a and carotenoids, together with polar lipid molecules, can reconstitute *in vitro* the fibrils observed in bell pepper fruit chromoplasts (Deruere *et al.*, 1994); in addition, overexpression of this fibrillin in tobacco led to an increase in the number of PGs organized into clusters in chloroplasts (Rey *et al.*, 2000). Considering these results, it was hypothesized that FBN1s may prevent PG coalescence and favor their clustering by acting as an interface between the aqueous environment and lipids, as well as by mediating cross-linkage via an unknown mechanism (Rey *et al.*, 2000). Our group has demonstrated interactions between Arabidopsis FBN1a and FBN1b proteins, as well as FBN1a–FBN1a and FBN1b–FBN1b interactions, providing a mechanism to explain the function of these FBNs in fibril formation or the maintenance of PG clusters (Gámez-Arjona *et al.*, 2014*a*). As noted above, FBN2 shows high homology to FBN1a and FBN1b, constituting a subgroup within the FBN family. Attempts to understand the function of this subgroup in Arabidopsis were made by Youssef *et al*. (2010) using an RNA interference strategy in which the expression of these three genes was reduced. This reduction led to pleiotropic effects, such as abnormal granal and stromal membrane arrangement, higher photosystem II (PSII) photoinhibition under stress, retarded shoot growth, and a deficit in anthocyanin accumulation under stress. These phenotypic alterations were alleviated by treatment with jasmonate (JA), and light/cold stress-related JA biosynthesis was suggested to be conditioned by the accumulation of PG-associated FBN1-2 proteins (Youssef *et al.*, 2010), although the mechanism explaining the function of the FBNs remains unclear.

In this study, we evaluated the function of members of the FBN1-2 subgroup by analysing the phenotypes of *fbn2*, *fbn1a-fbn1b*, and *fbn1a-1b-2* knockout mutant lines. We show that, unlike FBN1s, FBN2 displays dual localization, is soluble in the stroma, and is associated with the membrane fraction. FBN2 interacts with FBN1s and with another FBN2 polypeptide, indicating that these proteins may form a network around PGs. FBN2 also interacts with other proteins, including the enzymes catalysing the first steps of the synthesis of JA. This interaction seems to be necessary for the correct function of these proteins.

## Materials and methods

### Plant material and growth conditions

*Arabidopsis thaliana* plants were cultivated in a growth chamber under the following different conditions: normal conditions, a 16h light/8h darkness photoperiod, 110 µmol m^−2^ s^−1^ light intensity, 22 °C day/20 °C night temperature regimen, and 70% humidity; high-light stress, a 16h light/8h darkness photoperiod, 600 µmol m^−2^ s^−1^ light intensity, 22 °C day/20 °C night temperature regime, and 70% humidity; cold stress, a 16h light/8h darkness photoperiod, 110 µmol m^−2^ s^−1^ light intensity, 10 °C temperature, and 70% humidity; and cold and high-light stress, a 16h light/8h darkness photoperiod, 600 µmol m^−2^ s^−1^ light intensity, 10 °C temperature, and 70% humidity. The *fbn1a-1b* double mutant was described previously (Gámez-Arjona *et al.*, 2014*b*) and the *fbn2* (SALK_124590) mutant was obtained from the Salk T-DNA Mutants Collection (Alonso, 2003). A line with three mutations, *fbn1a-1b-2*, was obtained by crossing and selecting homozygous triple mutant plants from the segregating F_2_ population via PCR-based genotyping. All the primers used are listed in Supplementary Table S1.

### Plasmid construction

The full-length ORFs of *FBN1a*, *FBN1b*, *FBN2*, *FBN4*, and *allene oxide synthase* (*AOS*) were cloned (without the stop codon) into the pDONR207 entry vector (Invitrogen, http://www.lifetechnologies.com) using a BP reaction (Invitrogen). After sequence verification, the inserts were transferred into the binary vectors pXNGW [containing the cDNA encoding the N-terminal part of yellow fluorescent protein (YFP)] or pXCGW [containing the cDNA encoding the C-terminal part of cyan fluorescent protein (CFP)] (Yuan *et al.*, 2013) for bimolecular fluorescence complementation (BiFC) assays using LR Clonase II (Invitrogen). This resulted in translational fusion between the ORFs and the YFP/CFP moieties driven by the CaMV 35S promoter. *FBN2* and *FBN4* cDNAs were also transferred to the vector pGWB5, which allows the expression of FBN2-GFP or FBN4-GFP translational fusion constructs under the control of the 35S promoter (Nakagawa *et al.*, 2007).

For the construction of a genomic DNA fragment consisting of 1kb of the promoter region and the coding sequence of *FBN2*, the *Agrobacterium* pPZP211 binary vector (Xiang *et al.*, 1999) was engineered to generate pPZP211OCS by the addition of the *octopine synthase* gene (*OCS*) terminator via the *Bam*HI and *Pst*I restriction sites. The *OCS* terminator fragment was amplified by PCR using the oligonucleotides described in Supplementary Table S1 and with the pHANNIBAL vector used as template. The *FBN2* genomic DNA fragment was amplified by PCR (Supplementary Table S1), sequenced and cloned into the *Acc*65I and *Eco*RI restriction sites of the pPZP211OCS plasmid. Supplementary Fig. S1A shows a map of the resulting plasmid.

### Arabidopsis transformation and selection of transgenic plants

The binary vector pZPZ211OCS_FBN2genomic containing the genomic DNA encoding *FBN2* and 1kb of its promoter region was introduced into the Agroba*cterium tumefaciens* C58 strain and used to transform the Arabidopsis *fbn2* mutant by the floral dip method (Clough and Bent, 1998). The transformed plants were selected for kanamycin resistance. Ten independent T_3_ homozygous plants were selected and two of them were analysed by immunoblotting using anti-FBN2 antibodies to confirm the presence of the FBN2 protein (Supplementary Fig. S1B).

### Transient expression in *Nicotiana benthamiana*

The *Agrobacterium*-mediated transient expression of the *FBN1a*, *FBN1b*, *FBN2*, *FBN4*, and *AOS* genes in *N. benthamiana* leaves was carried out as described by Gámez-Arjona *et al.* (2014*b*).

### Pigment determination

For determination of anthocyanin concentrations, pigments were extracted by incubating the leaf samples in 1ml of acidic (1% HCl) methanol overnight according to the method described by Rabino and Mancinelli (1986). The absorbance of the extracts, clarified by centrifugation, was measured at 530nm and 657nm, and the formula A_530nm_–0.2A_657nm_ was employed to determine the anthocyanin concentration.

### Confocal microscopy

A DM6000 confocal laser scanning microscope (Leica Microsystems, http://www.leica-microsystems.com) equipped with a ×63 water-immersion objective was used to examine protein localization by GFP fusion or protein–protein interaction in BiFC assays involving *N. benthamiana* mesophyll cells. GFP or YFP/CFP expression and chlorophyll autofluorescence were imaged by excitation with a 488nm argon laser and detection at 500–525nm and 630–690nm, respectively.

### Isolation of soluble and membrane fractions from chloroplasts

Chloroplast isolation from Arabidopsis rosette leaves was performed according to the procedure described by Koochak *et al* (2019). Isolated chloroplasts were ruptured osmotically in ice-cold shock buffer [25mM HEPES, pH 7.5, 40mM KCl, 7mM MgCl_2_, supplemented with 1mM phenylmethylsulfonyl fluoride and 10 µl ml^–1^ of Protease Inhibitor Cocktail (Sigma, P9599)]. The soluble and membrane fractions of the chloroplasts were isolated by ultracentrifugation at 100000 *g* for 1h at 4 °C. The pellet (membrane fraction) was resuspended in shock buffer to the same volume as the supernatant (soluble fraction).

### Immunoblot analysis

Proteins were transferred from an SDS-polyacrylamide gel to a polyvinylidene difluoride (PVDF) membrane by electroblotting using a Trans-Blot SD transfer cell (Bio-Rad, www.bio-rad.com) according to the manufacturer’s instructions. Blots were probed with rabbit anti-FBN2 (GenScript, www.genscript.com) at 1:5000 dilution, rabbit anti-FBN4 (GenScript) at 1:1000 dilution, rabbit anti-glutamine synthetase (Agrisera) at 1:5000 dilution, or chicken anti-psbA (Agrisera) at 1:8000 dilution, followed by horseradish peroxidase-conjugated goat anti-rabbit IgG serum (Bio-Rad) at 1:25000 dilution for anti-FBN2, anti-FBN4, and anti-GS, or conjugated goat anti-chicken IgG serum (Agrisera) at 1:10000 dilution for anti-psbA. The hybridization signal was detected using WesternBright Quantum (Advansta), and the chemiluminescence was visualized using a Chemidoc Imaging System (Bio-Rad) running Quantity One software (Bio-Rad).

### Co-immunoprecipitation analysis

Chloroplast isolation and thylakoid membrane purification were performed following the procedure described by Koochak *et al.* (2019) from 30g of leaves from plants cultivated under normal growth conditions. An aliquot of thylakoid membranes equivalent to 250 µg of protein was resuspended in 500 µl (final volume) of 100mM phosphate buffer (pH 7) and supplemented with Triton X-100 at a final concentration of 0.01% (v/v) to extract FBN2 from the membrane fraction. The sample was incubated at 4 °C for 30min and then centrifuged at 100000 *g* for 1h at 4 °C. The pellet was discarded and the supernatant was subjected to co-immunoprecipitation (Co-IP) analysis using a polyclonal specific antibody against the whole FBN2 protein (produced by GENSCRIPT) and a Dynabeads Co-Immunoprecipitation Kit (Life Technologies, http://lifetechnologies.com) following the manufacturer’s instructions.

### Protein sample preparation and LC-MS/MS analysis

Protein treatment and analysis was performed as described by Vowinckel *et al.* (2014), with some modifications. After Co-IP, protein samples were precipitated with acetone and the pellet was resuspended in a 0.2% solution of RapiGest (Waters, www.waters.com) in 0.05 M ammonium bicarbonate. Dithiothreitol at a final concentration of 5mM was added and the samples were incubated for 30min at 60 °C. Finally, iodoacetamide was added at a final concentration of 10mM and the samples were incubated for 30min at room temperature in the dark. Digestion was performed by incubating the solution with trypsin at a ratio of 1:40 (trypsin:protein) overnight at 37 °C. After digestion, the equivalent of 1 µg of protein was analysed on a Tandem Quadrupole Time-of-Flight mass spectrometer (AB/Sciex TripleTOF5600 Plus) coupled with a Nanospray III Ion Source (AB/Sciex) and nano-HPLC (EKsigent Ultra 2D). Peptide separation was carried out by removing impurities on an isocratic pre-column (C18 PepMap100 column NAN75-15-03-C18-PM, Thermo Fisher Scientific) using 0.1% formic acid and 5% (v/v) acetonitrile as the solvent at a flow rate of 3 µl min^−1^ for 10min. Peptides were then eluted on to the analytical column using the incorporated electrospray emitter (New Objective PicoFrit column, 75 µm internal diameter × 250mm, packed with Reprosil-PUR 3 µm) and separated on a linear gradient of 5–35% solvent B for 60min at a flow rate of 250 nl min^−1^. Solvent A was 0.1% (v/v) formic acid, and solvent B was acetonitrile with 0.1% (v/v) formic acid. The ion source was operated with the following parameters: ISVF=2600, GS1=20, CUR=25. The data acquisition mode, using the DDA method, was set to obtain a high-resolution TOF-MS scan over a mass range of 400–1250 m/z, followed by MS/MS scans of 50 ion candidates per cycle (scan mass range 230–1500 m/z) with dynamic background subtraction, operating the instrument in high-sensitivity mode. The ion accumulation time was set to 250ms (MS) or 65ms (MS/MS). The proteins were identified using the software ProteinPilot v5.0.1 (Sciex) with the Paragon method, and the Arabidopsis proteome database (www.uniprot.org) in FASTA format fused to the contaminants of Sciex.

### Photosynthetic parameters

For analysis of chlorophyll *a* fluorescence, the plants were acclimated to the dark for 30min before making measurements. Chlorophyll *a* fluorescence was monitored using a Walz MAXI-IMAGING-PAM chlorophyll fluorometer. A pulsed blue measuring beam (1 Hz, intensity 4) was used to obtain the *F*_0_ value. Saturation pulses of 2700 µmol m^−2^ s^−1^ were applied for 0.8s to determine *F*_m_ and *F*_m_ʹ. The maximum quantum yield of PSII was calculated as *F*_v_/*F*_m_=(*F*_m_–*F*_0_)/*F*_m_. All measurements were obtained in fully developed leaves.

## Results

### Isolation of a knockout mutant lacking FBN2

**Fig. 1.**
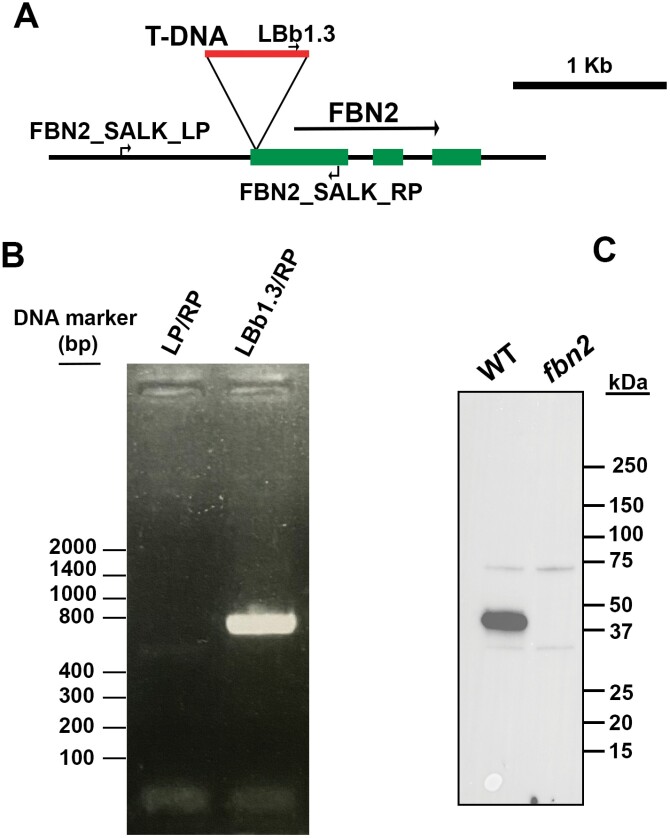
Isolation of the *fbn2* knockout mutant. (A) Schematic illustration of the genomic DNA fragment containing the *FBN2* gene. The T-DNA insertion in the first exon of *FBN2* found in the SALK_124590 line is shown. The primers used in the analysis of wild-type (WT) or mutant *FBN2* alleles are indicated. (B) PCR analysis of a homozygous *fbn2* mutant plant. Only the mutant allele is detected. (C) Immunoblot analysis of leaf extracts of WT and *fbn2* mutant plants using anti-FBN2 antibodies.

Searching in the different Arabidopsis mutant collections by T-DNA insertion revealed the line SALK_124590, which contains a T-DNA insertion in the first exon of the *FBN2* gene. A plant homozygous for the mutant allele was identified via PCR, and immunoblot analysis using a specific antibody against FBN2 determined that this line lacks the FBN2 protein (Fig. 1). No other lines lacking FBN2 could be found in the collections we searched.

### FBN2 is localized in both the stroma and in association with membranes

**Fig. 2.**
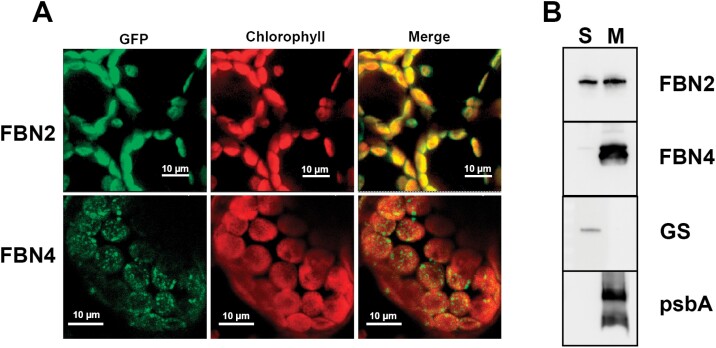
Localization of FBN2. (A) Full-length *FBN2* or *FBN4* cDNAs were fused to *GFP* and transiently expressed in *N. benthamiana* leaves. Fluorescence was monitored by confocal microscopy. The GFP fluorescence, chlorophyll autofluorescence, and merged images are shown. (B) Chloroplasts isolated from Arabidopsis rosette leaves were disrupted and the soluble (S) and membrane fractions (M) were isolated by ultracentrifugation at 100000 *g* for 1h at 4 °C. The pellet (membrane fraction) was resuspended in the same volume as the supernatant and 20 µg of protein from each fraction was loaded on to SDS-PAGE gels. The proteins were blotted on to a PVDF filter and hybridized with specific antibodies against FBN2, FBN4, plastidial glutamine synthetase (GS) (a marker of stromal protein), and psbA (a marker of thylakoid membranes).

Both FBN1a and FBN1b exhibit a dot-like pattern of localization when analysed using transient expression of FBN1-GFP fusion peptides in *N. benthamiana* leaves; this pattern is characteristic of proteins associated with PGs (Gámez-Arjona *et al.*, 2014*a*). However, FBN2-GFP exhibited a different pattern, and the detected fluorescence coincided with chlorophyll autofluorescence (Fig. 2A), indicating that FBN2 could be a soluble protein in the stroma or could be evenly associated with thylakoid membranes. As a control, we used the same technical approach to analyse the localization pattern of another FBN, FBN4, which has been described to be associated with PGs (van Wijk and Kessler, 2017). Unlike FBN2, FBN4 appeared to be localized in discrete patches throughout the chloroplast (Fig. 2A). It is possible that the GFP tagging of FBN2 could modify the localization pattern of the protein and produce an artifact. To ascertain the dual localization of FBN2, we carried out an immunoblot analysis of the soluble and membrane fractions from isolated chloroplasts. This analysis indicated that FBN2 is found both as a soluble protein in the chloroplast stroma and associated with membranes. In contrast, FBN4 was found to be exclusively associated with the membrane fraction (Fig. 2B). The localization of the plastidial glutamine synthetase (GLN2) or the D1 protein (PsbA) indicated the absence of cross-contamination between the two fractions. Given this analysis, we cannot ascertain whether the FBN2 protein associated with membranes is localized exclusively in association with PGs or could also be associated with other regions of the thylakoid membranes.

### Effect of cold and high-light stress on anthocyanin accumulation in plants with mutations in FBN1a, FBN1b, and FBN2

**Fig. 3.**
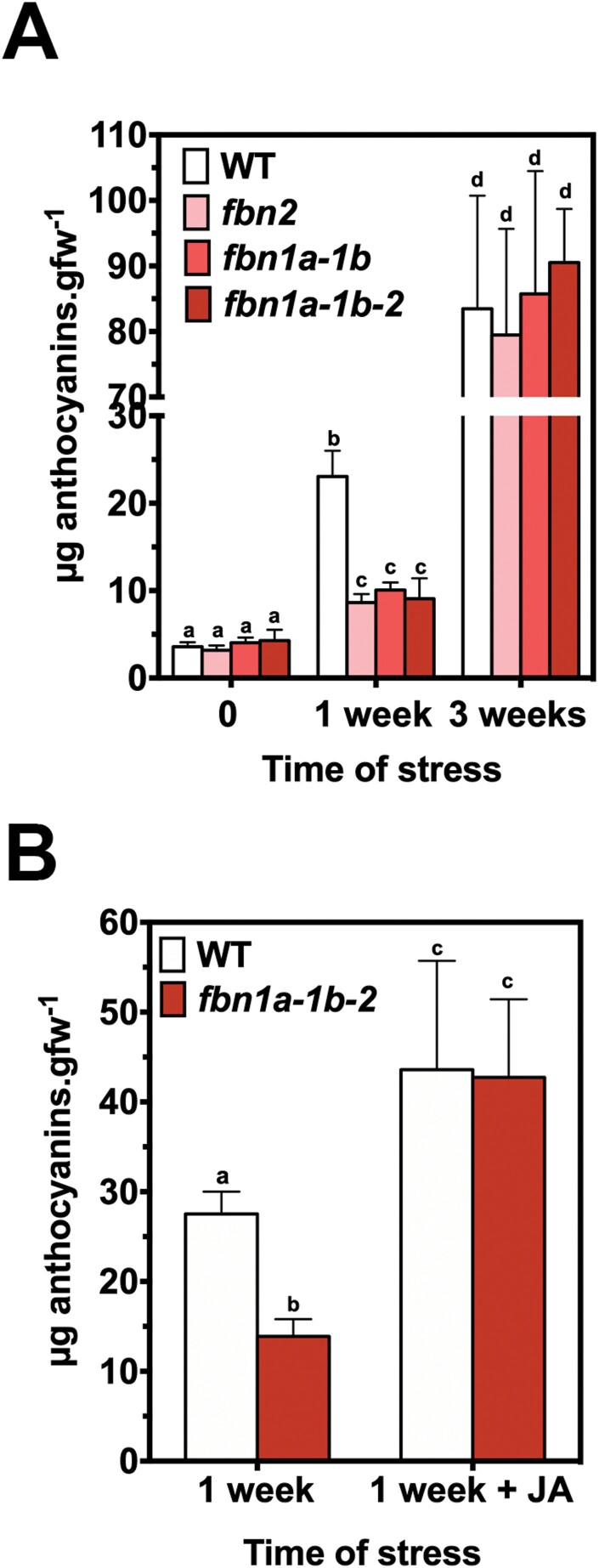
Anthocyanin accumulation in *fbn* mutants. (A) Knockout mutants *fbn2*, *fbn1a-1b*, and *fbn1a-1b-2* and WT plants were sown in soil and cultivated under normal conditions for 3 weeks. After this period, the plants were subjected to stress conditions (600 µmol m^−2^ s^−1^ light intensity and 10 °C temperature). Twelve rosette leaves from four plants per line were harvested after 0, 1, and 3 weeks of treatment, and the concentrations of anthocyanins were determined. (B) Plants cultivated under normal conditions were subjected to stress as described in (A) for 1 week with or without the addition of 2mM JA every 3 d, and the concentrations of anthocyanins were determined. In both panels, the values are presented as means ±SD. Two-way ANOVA was performed using PRISM software version 6.0. Tukey’s HSD test was used as a post hoc test. Significant differences (*P*<0.01) between mean values are indicated with different letters.

Previous studies have shown that *fbn1a-1b-2* knockdown mutant plants accumulate lower amounts of anthocyanins than wild-type (WT) plants when subjected to cold and high-light stress (Youssef *et al.*, 2010). To study the role of each protein in these phenotypic alterations, we analysed the anthocyanin accumulation in leaves of knockout mutants lacking FBN2 (*fbn2*), FBN1a and FBN1b (*fbn1a-1b*), or FBN2, FBN1a and FBN1b (*fbn1a-1b-2*). We did not analyse the single knockout mutants *fbn1a* and *fbn1b* because we have previously shown that these proteins can form homodimers and heterodimers (Gámez-Arjona *et al.*, 2014*a*), suggesting a high degree of functional redundancy between them. The combination of moderately high light (600 µmol m^−2^ s^−1^) and low temperature (10 °C) induced the accumulation of anthocyanins in the different lines. The induction was similar in the three mutant lines and considerably lower than the induction measured in WT plants after 1 week of stress (Fig. 3A). The decrease of the anthocyanin concentration found in the triple mutant *fbn1a-1b-2* was not accumulative with respect to the reduction observed in its parental lines *fbn1a-1b* and *fbn2*, suggesting that FBN2 and FBN1s act together to facilitate the accumulation of anthocyanins. Nevertheless, the levels of anthocyanins in the leaves of the different mutants reached the values detected in WT plants after 3 weeks of stress (Fig. 3A), indicating that the accumulation of anthocyanins was impaired, but not abolished, in the mutant lines. The delay in the accumulation of anthocyanins was abolished by treatment with JA (Fig. 3B), supporting the idea that this delay is a consequence of impaired synthesis of JA in the mutants.

### Effect of mutations on photosynthetic performance

**Fig. 4.**
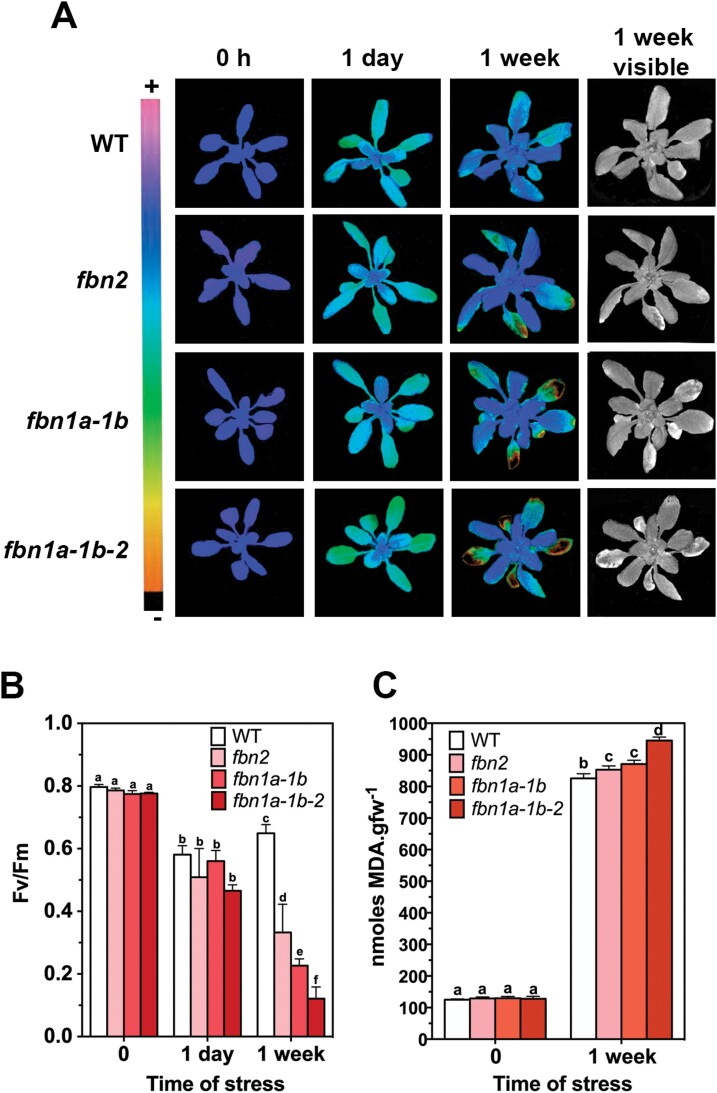
PSII performance of *fbn* plants. The plants were sown in soil, cultivated under normal conditions for 3 weeks, and then stressed for 1 week with a combination of moderate high light and low temperature as described in Fig. 3. (A) Representative false-colour images of WT, *fbn2*, *fbn1a-1b*, and *fbn1a-1b-2* plants at 0 d, 1 d, and 1 week of stress. The images represent the maximum PSII quantum yield (*F*_v_/*F*_m_). The last column shows the phenotypes of plants after 1 week of stress. (B) Six fully expanded leaves from two plants per line at 0h, 24h, and 1 week of stress were used to determine the *F*_v_/*F*_m_ values. (C) Concentrations of MDA in the leaves of 3-week-old plants subjected to high-light and cold stresses for 0h and 1 week. Four plants (three fully expanded leaves per plant) per line were used to determine the concentrations of MDA at each time point. In (B) and (C) the values are presented as means ±SD. PRISM software (version 6.0) was used to carry out a two-way ANOVA with Tukey’s post hoc test. Significant differences (*P*<0.01) between mean values are indicated with different letters.

One of the functions proposed for FBNs has been protection of the photosynthetic apparatus against damage induced by different abiotic stresses (Singh and McNellis, 2011). We analysed the performance of PSII by measuring *F*_v_/*F*_m_ in different mutant plants subjected to high light and cold stress. Fig. 4A shows the analysis of PSII chlorophyll *a* fluorescence, using a pulse-amplitude-modulated (PAM) fluorimeter (in this case we used an IMAGING-PAM device), of a representative plant of each line (WT, *fbn2*, *fb1a-1b*, and *fbn1a-1b-2*) subjected to moderate high-light and low-temperature stresses. A decrease in *F*_v_/*F*_m_ was observed in all the plants after 1 d of stress. This decrease was more evident in fully developed leaves than in young leaves. After 1 week of stress, all the plants showed evidence of acclimation, with higher *F*_v_/*F*_m_ values, especially in young leaves. However, the mutant lines showed lesions in some fully developed leaves, whereas such lesions were not detected in WT plants. The *F*_v_/*F*_m_ value in the lesioned leaves was considerably lower. The lesions were more evident in the triple mutant than in its parental lines *fbn1a-1b* and *fbn2*. Fig. 4B shows the quantification of *F*_v_/*F*_m_ parameter in six fully developed leaves of two plants per line. This analysis corroborates the finding that *F*_v_/*F*_m_ was lower in mature leaves of the *fbn2* mutant compared with the WT. The decrease was larger in *fbn1a-1b* and even larger in the triple mutant *fbn1a-1b-2*. The stress treatment applied (a combination of high light and low temperature) induces a strong quenching component, qH, which is dependent on the plastid lipocalin (Malnoë *et al.*, 2018). Changes of qH could explain the *F*_v_/*F*_m_ values observed in the different lines. These changes would be reflected in changes in the minimum (*F*_0_) and maximum (*F*_m_) fluorescence of the different plant lines. As shown in Supplementary Fig. S2, the *F*_0_ and *F*_m_ values were not statistically different between the plant lines (except the *F*_m_ of WT and *fbn1a-1b-2* plants after 1 week of stress), eliminating the possibility that the alteration of *F*_v_/*F*_m_ is a consequence of changes in qH in the mutant lines.

The lesions observed in the mutant lines may be a consequence of oxidative stress produced by the dysfunction of PSII, which could increase the ROS in these lines. To test this hypothesis, we measured the concentrations of malondialdehyde (MDA) as a marker of ROS-mediated lipid peroxidation. The concentrations of MDA were higher in the *fbn2* and *fbn1a-1b* lines than in the WT, and even higher in the f*bn2-fbn1a-fbn1b* triple mutant (Fig. 4C), supporting the idea that the lesions observed in the mutant lines are a consequence of stress-mediated increased levels of ROS.

### Complementation of the *fbn2* mutant

**Fig. 5.**
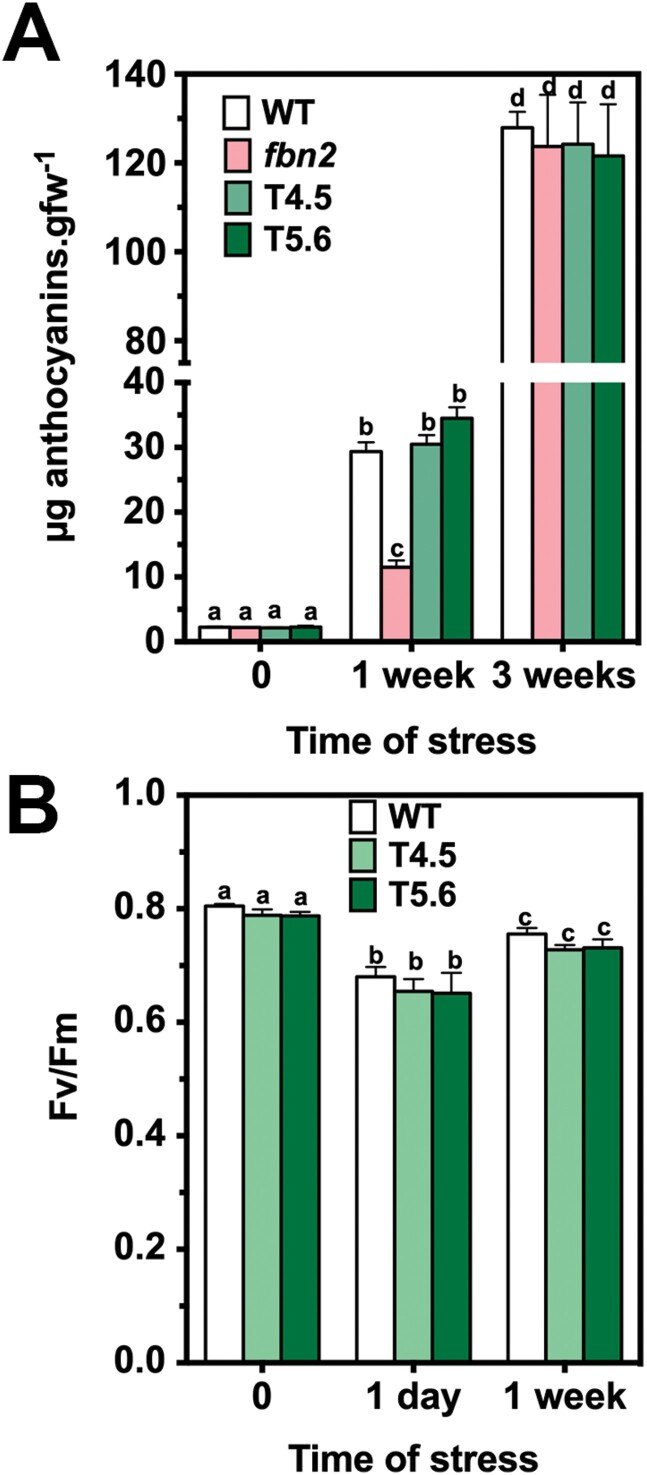
Accumulation of anthocyanins and *F*_v_/*F*_m_ of *fbn2* plants transformed with the *FBN2* gene. (A) WT, *fbn2*, and two transgenic plants transformed with a genomic DNA fragment containing the *FBN2* gene and 1kb of its promoter region (plants T4.5 and T5.6) were cultivated in a growth chamber under normal conditions and then subjected to stress conditions as described in Fig. 3. Twelve rosette leaves from four plants per line were harvested after 0, 1, and 3 weeks of treatment, and the concentrations of anthocyanins were determined. (B) WT, T4.5, and T5.6 plants grown under normal conditions were subjected to high-light and cold stresses, and the *F*_v_/*F*_m_ values of six fully expanded leaves from two plants per line were determined at 0 d, 1 d, and 1 week of treatment using an IMAGING-PAM device. In both panels, the values are presented as means ±SD. PRISM software (version 6.0) was used to carry out a two-way ANOVA with Tukey’s post hoc test analysis. Significant differences (*P*<0.01) between mean values are indicated with different letters.

As noted above, no other mutant line lacking FBN2 was found in the different mutant collections. To determine whether the phenotypic alterations found in *fbn2* mutant plants are a consequence of the lack of FBN2, we proceeded to complement the mutation by transforming the *fbn2* mutant with a genomic fragment containing the WT version of the FB2 gene together with 1kb of its promoter region (Supplementary Fig. S1A). Two independent transgenic lines expressing the FBN2 protein, T4.5 and T5.6 (Supplementary Fig. S1B), were selected for further studies. The anthocyanin concentrations of the transgenic plants after 1 or 3 weeks of a moderate combination of high light and low temperature were the same as those measured in the WT plants (Fig. 5A). In addition, IMAGING-PAM analysis confirmed that the *F*_v_/*F*_m_ of the two transgenic lines was similar to that of the WT (Fig. 5B). Taken together, these data indicate that the alterations observed in *fbn2* were due to the lack of the FBN2 protein.

### Screening for proteins that interact with FBN2

We have previously shown that FBN1a and FN1b interact with other proteins (Gámez-Arjona *et al.*, 2014*a*). Therefore, we analysed whether FBN2 could also interact with other polypeptides, which might explain the alterations observed in the *fbn2* mutant. We performed Co-IP assays using a specific polyclonal antibody against the full-length FBN2 protein.

As described above, FBN2 exhibited both stromal and membrane-associated localization. Thus, we decided to immunoprecipitate FBN2 in both fractions. In the case of the FBN2 protein associated with PGs, it was possible that precipitation of this protein could co-purify other proteins that are associated with these organelles but do not interact with FBN2. To avoid this artefact, after separation of the soluble and membrane fractions, we solubilized FBN2 from PGs by incubating the samples with 0.01% (v/v) Triton X-100, the concentration of this non-ionic detergent that solubilizes FBN2 from PGs (Supplementary Fig. S3), before Co-IP. We checked that the anti-FBN2 antibody does not recognize the FBN1a or FBN1b proteins by immunoblotting under native and denaturing conditions (Supplementary Fig. S4). Finally, a parallel analysis was performed using *fbn2* plants to identify proteins that co-immunoprecipitated in a non-specific manner. Proteins obtained by Co-IP were identified by mass spectrometry, and those also found in *fbn2* extracts were removed (these were mainly highly abundant proteins from the photosystems and light-harvesting complexes) (Supplementary Datasets S1–S8). We performed three independent Co-IP experiments and the proteins found in all replications are listed in Table 1 (FBN2 associated with PGs). In the case of the soluble population of FBN2, only isoforms 1 and 2 of fructose bisphosphate aldolase (FBA) were found by Co-IP. These isoforms have been described as proteins associated with PGs (Vidi *et al.*, 2006; Ytterberg *et al.*, 2006) but are not considered part of the core proteome (Lundquist *et al.*, 2012). The rest of the proteins that were identified seemed to interact exclusively with the FBN2 population associated with PGs. Some of these proteins have been described previously as components of the core proteome of PGs (Lundquist *et al.*, 2012). Interestingly, the ABC1-type kinases, the second most abundant type of protein in PGs, were not found in these analyses, indicating that the identified proteins bind to FBN2 and were not simply contaminants of PG-associated proteins.

**Table 1.** Proteins that co-immunoprecipitated with FBN2

AGI code	Name	Previously described on PGs
At4g04020	**Fibrillin 1a (FBN1a)**	**Yes** ^a^
At2g21330	Fructose bisphosphate aldolase 1	Yes^b^
At4g38970	Fructose bisphosphate aldolase 2	Yes^b^
At5g42650	Allene oxide synthase (AOS)	Yes^b^
At5g38660	Acclimation of photosynthesis to environment (APE1)	No
At4g19170	**9-*cis*-epoxycarotenoid dioxygenase 4 (CCD4)**	**Yes** ^a^
At1g20020	Ferredoxin-NADP[H]-oxidoreductase 2 (FNR2)	No
At3g18890	NAD(P)-binding Rossmann-fold superfamily protein (Tic62)	No
At3g26840	**Diacylglycerol acyltransferase 4 (DGAT4)**	**Yes** ^a^
At5g08740	**NAD(P)H dehydrogenase C1**	**Yes** ^a^
At4g23890	NAD(P)H-quinone oxidoreductase subunit S (NdhS)	No
At1g32220	**Flavin-reductase-related 1**	**Yes** ^a^
At4g35250	NAD(P)-binding Rossmann-fold superfamily protein	No

The proteins identified by Lundquist *et al.* (2012) as part of the core PG proteome are highlighted in bold.

Lundquist *et al.* (2012)

Vidi *et al.* (2006); Ytterberg *et al.* (2006)

Isoforms 1 and 2 of FBA were also identified to interact with the FBN2 fraction associated with PGs. Finally, other proteins we identified have not been previously described as being associated with PGs, such as isoform 2 of the ferredoxin-NADP[H] oxidoreductase (FNR2), which forms a complex with Tic62 (also found by Co-IP) (Benz *et al.*, 2009), or the APE1 protein, which is involved in adaptation of the plant, specifically the thylakoid membranes, to fluctuating environmental conditions (Walters *et al.*, 2003).

### Interaction of FBN2 with FBN1s and other FBN2 polypeptides

**Fig. 6.**
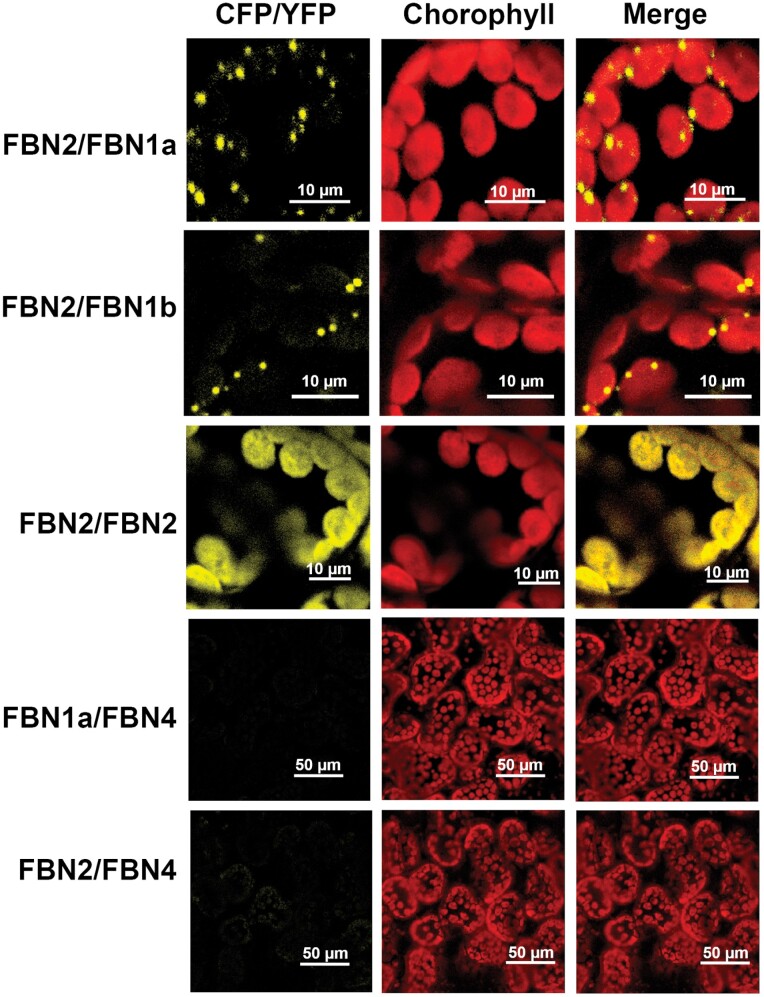
*In vivo* interaction of FBN2–FBN1a, FBN2–FBN1b, FBN2–FBN2, FBN2–FBN4, and FBN1a–FBN4. cDNAs encoding the full-length FBN1a or FBN2 proteins were fused to the N-terminal half of YFP and co-transformed into *N. benthamiana* leaves together with cDNAs encoding FBN2, FBN1a, FBN1b, or FBN4 fused to the C-terminal moiety of CFP. The images show the YFP/CFP (BiFC) fluorescence, the chlorophyll autofluorescence, and the merged images.

One of the proteins that co-immunoprecipitated with FBN2 was FBN1a (Table 1). The possible interaction between these two proteins was confirmed by BiFC assays during transient expression in *N. benthamiana* leaves. Fig. 6 shows the *in vivo* interaction of FBN2 with FBN1a, presenting a dot-like pattern similar to that observed for the SS4–FBN1a (Gámez-Arjona *et al.*, 2014*b*) or FBN1b–FBN1a (Gámez-Arjona *et al.*, 2014*a*) interactions. A similar pattern was also observed for the interaction between FBN2 and FBN1b. Considering the homology between these proteins, we analysed whether FBN2 could also bind to another FBN2 polypeptide. This interaction was confirmed *in vivo* when FBN2 was expressed transiently in *N. benthamiana* leaves (Fig. 6). The specificity of these interactions was determined by using the FBN4 polypeptide as a negative control. Together with the FBN1-2 subfamily, this protein is the most abundant protein associated with PGs. BiFC analysis of FBN4–FBN1a and FBN4–FBN2 indicated that these proteins do not interact, although they are all localized to PGs (Fig. 6).

### FBN2 binds to allene oxide synthase

**Fig. 7.**
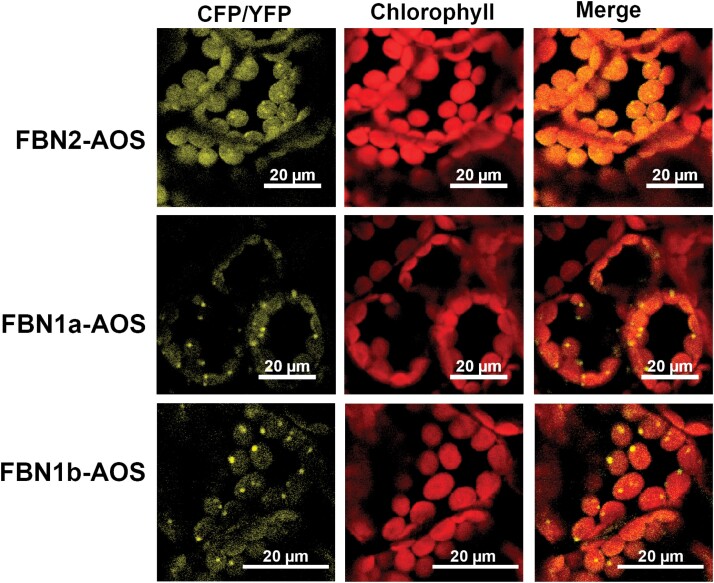
*In vivo* interaction of FBN2–AOS, FBN1a–AOS, and FBN1b–AOS. cDNAs encoding the full-length FBN1a, FBN1b, or FBN2 proteins were fused to the N-terminal half of YFP and co-transformed into *N. benthamiana* leaves together with cDNA encoding AOS fused to the C-terminal moiety of CFP. Images show the YFP/CFP (BiFC) fluorescence, the chlorophyll autofluorescence, and the merged images.

Co-IP analysis revealed AOS to be a protein that binds to FBN2. This protein has been previously described to be associated with PGs and, together with lipoxygenase (LOX) and allene oxide cyclase (AOC), catalyses the first steps of the biosynthesis of JA in the chloroplast (Wasternack and Song, 2017). Lipoxygenase 2 (LOX2) and AOC were found in these analyses but were not included in Table 1 because they were not detected in all three replications. As shown in Fig. 7, AOS can interact *in vivo* with FBN2, confirming the results obtained in the Co-IP analysis. In addition, we found that AOS is able to interact with FBN1a or FBN1b *in vivo* (Fig. 7).

## Discussion

FBNs have been proposed to be involved in central processes of plant physiology, such as plant responses to stress and the development of plastid architecture (Singh and McNellis, 2011). FBN1a, FBN1b, FBN2, and FBN4 are the most abundant proteins in PGs and have been identified as component of the PG core proteome. FBN2 shows a PG/stroma abundance ratio of 1188 (Lundquist *et al.*, 2012), indicating that most FBN2 protein is associated with PGs. However, analysis of FBN2-GFP localization suggests that this protein is also present in the stroma or uniformly localized in the thylakoid membranes. Immunoblot analysis of the plastidial soluble and membrane fractions indicated that FBN2 was distributed in similar proportions in these two fractions. The presence of two populations of FBN2 with different localizations could suggest a post-translational modification of the protein or the existence of splice variants. In this respect, it is worth noting the work of Lohscheider *et al.* (2016), which identified the presence of two phosphorylation sites in FBN2. However, FBN4 showed five phosphorylation sites (Lohscheider *et al.*, 2016) but it was found exclusively in the membrane fraction (Fig. 2B), suggesting that phosphorylation is not responsible for the dual localization of FBN2. We could not determine whether the membrane-associated population of FBN2 is localized exclusively in PGs, and it could also be localized in other regions of the thylakoid membranes. The different methodologies employed—mass spectrometry in the analysis of Lundquist *et al.* (2012) and GFP fusion and immunoblotting in this study—may explain the discordance between the results of the two analyses. These results are also different from those obtained from the analysis of FBN4-GFP (Fig. 2) and FBN1-GFP (Gámez-Arjona *et al.*, 2014*b*), which observed a dot-like pattern of localization characteristic of PG-associated proteins, and it is the first evidence pointing to different functions of FBN2 and FBN1s.

The elimination of FBN1s or FBN2 slowed the high-light-induced accumulation of anthocyanins in the mutant plants (Fig. 3A). The suppression of this alteration when plants are treated with JA indicates that the elimination of FBN2 or FBN1s affects the synthesis of JA (Fig. 3B). We have shown that FBN2 or FBN1a interact with AOS (Fig. 7), which catalyses one of the first steps of JA synthesis. FBN2 likely also interacts with LOX2 and with AOC, as found in some Co-IP experiments. AOS was previously identified to be a protein associated with PGs (Vidi *et al.*, 2006; Ytterberg *et al.*, 2006), although Lundquist *et al.* (2012) did not consider it to be part of the PG core proteome. In addition, recruitment of the three plastidial enzymes (LOX, AOS, and AOC) involved in the synthesis of JA to the PGs of stressed plants has been shown (Lundquist *et al.*, 2013). Recently, the existence of a chloroplast envelope complex comprising LOX2, AOS, and AOC was described (Pollmann *et al.*, 2019). These findings suggest the existence of two populations of enzymes, one that localizes to the chloroplast envelope and another that associates with PGs. Our data suggest that FBN1s and FBN2 mediate the association of these enzymes with PGs, facilitating the flux of metabolites through them. The slowed accumulation of anthocyanins would be more noticeable in *fbn* mutants subjected to abiotic stresses, a situation that increases the number of PGs in the cells (van Wijk and Kessler, 2017) and the amount of FBNs (Ytterberg *et al.*, 2006). The recruitment of enzymes involved in JA synthesis to the PGs would be affected by the elimination of FBN2 or FBN1s. Nevertheless, we cannot discount that the interaction of these FBNs with the JA synthesis-related enzymes positively modulates the activity of these enzymes.

**Fig. 8.**
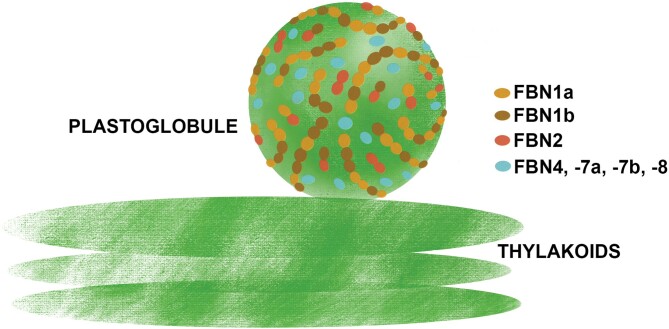
Schematic model of the arrangement of FBNs1-2 subgroup proteins on the surface of PGs. FBN2 (red) may form homodimers or heterodimers with FBN1a (light brown) or FBN1b (dark brown). We have previously shown that FBN1a and FBN1b may form hetero-oligomers (Gámez-Arjona *et al*, 2014*a*). These interactions allow the formation of a FBNs1-2-based network around the surface of PGs. Other proteins, such as those described in Table 1, associate with PGs via interactions with these FBNs. The degree of functional redundancy between these FBNs has not been characterized and might vary for each PG-associated protein. Their elimination would affect the localization and function of some PG-associated proteins. The functions of other FBNs associated with PGs (FBN4, FBN7a, FBN7b, and FBN8, indicated in light blue) have not been determined yet.

The function of these FBNs in JA synthesis does not seem to be redundant, as the same alteration was observed in the *fbn1a-1b*, *fbn2*, and *fbn1a-1b-2* mutants, indicating that FBN1s and FBN2 are necessary for this process and the elimination of any of them alters the synthesis of JA. In this sense, the interaction observed between FBN2 and FBN1s is interesting. We previously showed that, by interacting with each other, FBN1a and FBN1b can form homopolymers or heteropolymers *in vivo* (Gámez-Arjona *et al.*, 2014*a*). These findings suggest that these three FBNs may form a net on the surface of PGs, and we speculate that this net is necessary for the recruitment of the enzymes involved in JA synthesis to PGs, so that the elimination of FBN1s or FBN2 would alter JA synthesis in stressed plants. Fig. 8 depicts a model of the possible arrangement of these FBNs on the surface of PGs.

Another phenotypic alteration observed in the *fbn1-2* mutants is the presence of necrotic tissues in fully expanded leaves of plants subjected to high light and cold stress. These lesions were more abundant in the *fbn1a-1b-2* triple mutant than in its parental lines, *fbn1a-1b* and *fbn2* (Fig. 4A). Analysis of the performance of PSII in the mutant plants subjected to these stresses indicated that the maximum quantum yield of PSII is lower in *fbn1a-1b* and *fbn2* than in the WT, and it is even lower in the triple mutant *fbn1a-1b-2* (Fig. 4B). The low efficiency of PSII in the mutant lines may lead to an increase in ROS levels, which would be responsible for programmed cell death and, hence, the presence of necrotic tissues (Triantaphylidès *et al.*, 2008). One consequence of ROS formation is lipid peroxidation (Montillet *et al.*, 2005), and MDA is a marker for this free-radical-catalysed peroxidation (Farmer and Mueller, 2013). The concentrations of MDA were higher in the *fbn1a-1b* and *fbn2* plants than in the WT, and even higher in *fbn1a-1b-2* (Fig. 4C), supporting the idea that the lesions found in the *fbn1-2* mutants are a consequence of stress-mediated ROS formation in these lines. Unlike the accumulation of anthocyanins, the FBN1-2 protection of PSII against high light and cold stress is additive: the lesions found in the *fbn1a-1b-2* mutant were more abundant than those in *fbn1a-1b* or *fbn2* (Fig. 4A) and the *F*_v_/*F*_m_ values and MDA concentration were lower and higher, respectively, in the triple mutant than in its parental lines (Fig. 4B, C). These results suggest that these FBNs show a degree of redundancy in the protection of PSII against this type of stress, although it cannot be discounted that they act through different mechanisms.

Co-IP analyses indicated that FBN2 not only interacts with FBN1a and AOS, but also interacts with many other proteins (Table 1). Some of these proteins, such as FNR2, NAD(P)H dehydrogenase C1, the S subunit of the NDH complex, or APE1, are closely related to photosynthesis. It is tempting to speculate that, as for the JA biosynthesis-related proteins, FBN2 may facilitate the correct positioning of these proteins. The elimination of FBN2 would lead to dysfunction of the interacting proteins and produce different pleiotropic effects, including increased damage to PSII under high light and cold stress. Further analyses are needed to ascertain this possibility.

As noted above, unlike FBN1a or FBN1b, FBN2 displays a dual localization pattern, and it is present both in the stroma and associated with membranes. The results of the Co-IP analysis suggest that the membrane-associated population of FBN2 is a more active form than the soluble population, as only two proteins were found to bind to the soluble form of FBN2 protein, and these proteins also interacted with the membrane-associated form of FBN2. These proteins are the most abundant plastidial isoforms of FBA in leaves; the other plastidial isoform is mainly expressed in roots (Lu *et al.*, 2012). FBA catalyses the condensation of fructose-1,6-biphosphate and the condensation of sedoheptulose-1,7-biphosphate in the Calvin–Benson cycle in plastids. Ytterberg *et al.* (2006) found these proteins in an analysis of the PG proteome and suggested that, as no other enzymes from the Calvin–Benson cycle were found, these aldolases made an unknown functional contribution to the metabolism and/or structure of PGs (Ytterberg *et al.*, 2006). Further characterization is necessary to understand the physiological meaning of the FBA–FBN2 interactions and determine the mechanisms responsible for the partitioning of FBN2 into two compartments.

## Supplementary data

The following supplementary data are available at *JXB* online

Dataset S1. Co-immunoprecipitation results in *fbn2* mutant (membrane fraction); replicate 1.

Dataset S2. Co-immunoprecipitation results in *fbn2* mutant (membrane fraction); replicate 2.

Dataset S3. Co-immunoprecipitation results in *fbn2* mutant (membrane fraction); replicate 3.

Dataset S4. Co-immunoprecipitation results in *fbn2* mutant (stroma fraction).

Dataset S5. Co-immunoprecipitation results in WT (membrane fraction); replicate 1.

Dataset S6. Co-immunoprecipitation results in WT (membrane fraction); replicate 2.

Dataset S7. Co-immunoprecipitation results in WT (membrane fraction); replicate 3.

Dataset S8. Co-immunoprecipitation results in WT (stroma fraction).

Table S1. Oligonucleotides used in this work.

Fig. S1. Map of pPZP211OCS_FBN2 genomic plasmid and immunoblot analysis of transformed *fbn2* plants.

Fig. S2. *F*_0_ and *F*_m_ values of WT and *fbn2*, *fbn1a-1b*, and *fbn1a-1b-2* mutant plants.

Fig. S3. Solubilization of FBN2 by Triton X-100 treatment.

Fig. S4. Specificity of anti-FBN2 antibody.

erab452_suppl_Supplementary_Dataset_S1

erab452_suppl_Supplementary_Dataset_S2

erab452_suppl_Supplementary_Dataset_S3

erab452_suppl_Supplementary_Dataset_S4

erab452_suppl_Supplementary_Dataset_S5

erab452_suppl_Supplementary_Dataset_S6

erab452_suppl_Supplementary_Dataset_S7

erab452_suppl_Supplementary_Dataset_S8

erab452_suppl_Supplementary_Table_S1_Figures_S1-S4

## Data Availability

All data supporting the findings of this study are available within the paper and within its supplementary data published online
