# Supplementary material for: Arabidopsis fibrillin 1-2 subfamily members exert their functions via specific protein–protein interactions
Source: J Exp Bot. 2021 Oct 15;73(3):903–14. doi: 10.1093/jxb/erab452 (PMC8793873; doi:10.1093/jxb/erab452)
Supplement: erab452_suppl_Supplementary_Table_S1_Figures_S1-S4 [file erab452_suppl_supplementary_table_s1_figures_s1-s4.pdf]

Supplementary Table S1. Oligonucleotides used in this work

Name	Gene	Sequence	Use
FBN1a_F	FBN1a (At4g04020)	CTATCCAGGCCCATTTAGTCC	Oligos used to amplify the WT allele in the T-DNA mutant line SALK_024528
FBN1a_R		TCACCGGGAAATTAAACTTCC	
FBN1b_F	FBN1b (At4g22240)	AAGAACCCTACTCCAGCTCCGA	Oligos used to amplify the WT allele in the T-DNA mutant line Sail_384_a10
FBN1b_R		TCAAGGATTCAAGAGAGGGCTT	
FBN2_SALK_LP	FBN2 (At2g35490	TGTAATCAGCAATCCATTCCG	Oligos used to amplify the WT allele in the T-DNA mutant line SALK_124590
FBN2_SALK_RP		TTCAGTTCCGTACACCGAATC	
LBb1.3	T-DNA insert	ATTTTGCCGATTTCGGAAC	To identify mutant alleles in the T-DNA SALK lines together with FBN1a_R (*fbn1a* mutant) and FBN2_SALK_RP (*fbn2* mutant)
SynLB3		TAGCATCTGAATTTCATAACCAATCTCGAT	To identify mutant alleles in the T-DNA Syngenta line (*fbn1b* mutant) together with FBN1b_R
FBN1a_attB1	FBN1a (At4g04020)	GGGGACAAGTTTGTACAAAAAAGCAGGCTTC ATGGCGACGGTACCATTGTTCACC	Cloning of FBN1a full-length cDNA into pDONR207 (without the stop codon)
FBN1a_attB2		GGGGACCACTTTGTACAAGAAAGCTGGGTCAAC AGGGTTTAAGAGAGAGCTTCCTTC	

FBN1b_attB1	FBN1b (At4g22240)	GGGGACAAGTTTGTACAAAAAAGCAGGCTTC ATGGCGACGGTACAATTGTCC	Cloning of FBN1b full-length cDNA into pDONR207 (without the stop codon)
FBN1b_attB2		GGGGACCACTTTGTACAAGAAAGCTGGGTCA GGATTCAAGAGAGGGCTTCC	
FBN2_attB1	FBN2 (At2g35490)	GGGGACAAGTTTGTACAAAAAAGCAGGCTTCAT GGCTACGCTCTTCACCGTC	Cloning of FBN2 full-length cDNA into pDONR207 (without the stop codon)
FBN2_attB2		GGGGACCACTTTGTACAAGAAAGCTGGGTAGA GCTCAAGCAGAGAGCTTCC	
FBN4_attB1	FBN4 (At3g23400)	GGGGACAAGTTTGTACAAAAAAGCAGGCTTCAT GGCGACTTCTTCTACTTTC	Cloning of FBN4 full-length cDNA into pDONR207 (without the stop codon)
FBN4_attB2		GGGGACCACTTTGTACAAGAAAGCTGGGTAAGC AATGACGAATACCCTAAG	
AOS_attB1	AOS (At5g42650)	GGGGACAAGTTTGTACAAAAAAGCAGGCTTCA TGGCTTCTATTTCAACCCCT	Cloning of AOS full-length cDNA into pDONR207 (without the stop codon)
AOS_attB2	AOS (At5g42650)	GGGGACCACTTTGTACAAGAAAGCTGGCGAAA AGCTAGCTTTCCTTAA	
OCSterm_F	Octopine synthase	GGATCCTCTAGAGTCCTGCTTTAA	Cloning of OCS terminator in pPZP211
OCSterm_R		GTCGAGGCTCAGCAGGACCTGCAG	
FBN2g_F	FBN2 (At2g35490)	GAATTCCCACTTCAATTCCCCATCCACCTTCB	Cloning of the genomic DNA fragment containing FBN2 and its promoter in pZPZ211
FBN2g_R		GGTACCTCAGAGCTCAAGCAGAGAGCTTCC	

Supplementary Fig. S1

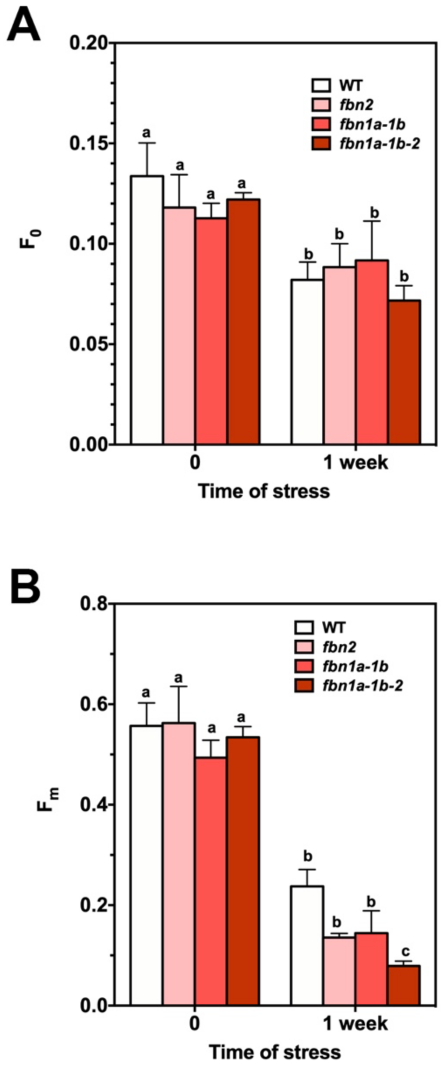

Figure S1. F_0 and Fm values of WT, *fbn2*, *fbn1a-1b*, and *fbn1a-1b-2* mutant plants. These values were obtained from the same experiment indicated in Figure 4. The values represent the mean ± SD. PRISM software (version 6.0) was used to carry out a two-way ANOVA with Tukey's *post hoc* test analysis. Significance is denoted if $p<0.01$, and mean values varying significantly at the 1% level are marked with different letters.

Supplementary Fig. S2

A

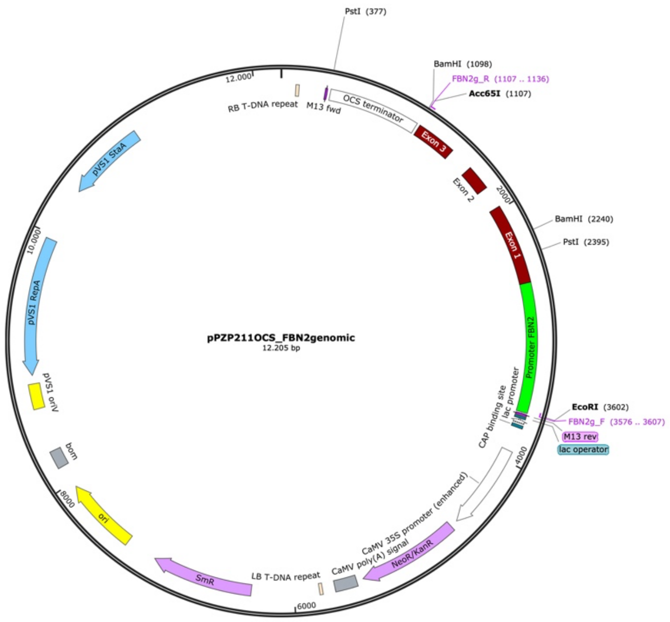

B

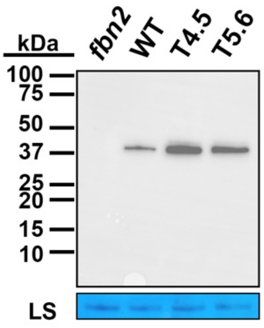

Figure S2. Complementation of *fbn2* mutation. **A**. Map of the binary plasmid used to transform to *fbn2* mutant plants. The three exons of FBN2 are indicated in red. The promoter region of FBN2 (1 kbp) is indicated in green. Restriction sites used to clone the OCS terminator (PstI/BamHI) and the FBN2 genomic DNA (Acc65I/EcoRI) in pPZP211 are indicated. **B**. Immunoblot analysis of *fbn2*, WT, transgenic T4.5 and transgenic T5.6 plants. Leaves crude extracts of these plants (15 µg) were loaded into a SDS-PAGE, blotted onto a PVDF membrane and hybridized with specific antibody against FBN2. After the chemiluminescence detection, the filter was stained with Coomassie-blue and the large subunit (LS) of Rubisco is shown as loading control

Supplementary Fig. S3

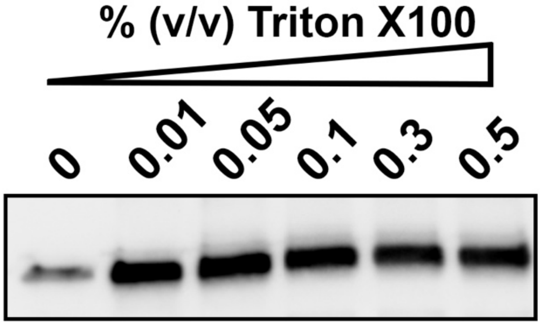

Figure S3. Solubilization of FBN2 by Triton X-100 treatment. Chloroplasts isolated from *Arabidopsis* rosette leaves were disrupted and the resulting extract treated with increasing amounts (v/v) of Triton X100 and incubated at 4ºC for 30 min. After the incubation, samples were ultracentrifuged at 100,000 x g for 1 hour at 4ºC. The pellet was discarded and 20 µl of each soluble fraction run in SDS-PAGE. Proteins were blotted on a PVDF filter and hybridized with specific antibodies against FBN2

Supplementary Fig. S4

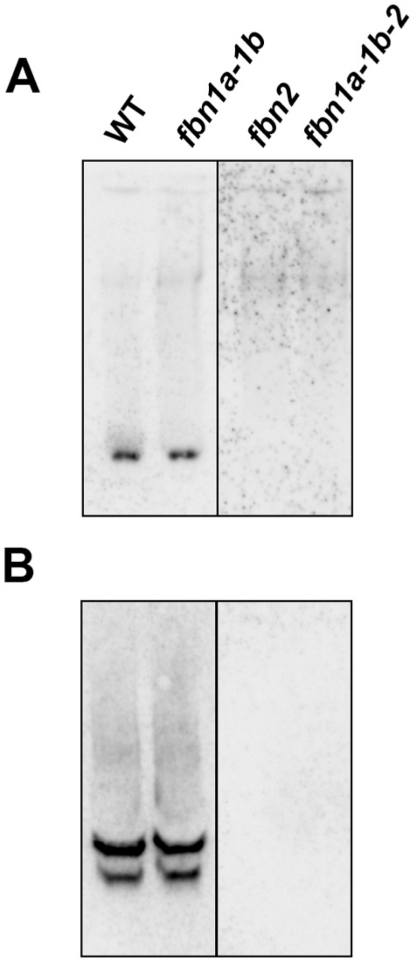

Figure S4. Specificity of anti-FBN2 antibody. Rosette leaves from *Arabidopsis* plants cultured under normal growth conditions were disrupted with a Tissue Tearor (Biospec Products Inc., Bartlesville, OK, USA) in 50 mM Hepes (pH 7.5) buffer, and 25 µg of protein from the whole crude extracts were loaded into a native (**A**) or SDS (**B**) polyacrylamide electrophoresis gel. Proteins were transferred to a PVDF filter and hybridized with anti-FBN2
